# Associations between societal disapproval and changes in symptoms of PTSD and appetitive aggression following treatment among high-risk South African males

**DOI:** 10.1080/20008198.2017.1369831

**Published:** 2017-09-05

**Authors:** Jessica Sommer, Martina Hinsberger, Leon Holtzhausen, Debra Kaminer, Soraya Seedat, Thomas Elbert, Mareike Augsburger, Andreas Maercker, Roland Weierstall

**Affiliations:** ^a^ Department of Psychology, University of Konstanz, Konstanz, Germany; ^b^ Department of Social Development, University of Cape Town, Cape Town, South Africa; ^c^ Department of Psychology, University of Cape Town, Cape Town, South Africa; ^d^ Department of Psychiatry, Stellenbosch University, Stellenbosch, South Africa; ^e^ Department of Psychopathology and Clinical Intervention, University of Zurich, Zurich, Switzerland

**Keywords:** Violence, social acknowledgment, posttraumatic stress disorder, appetitive aggression, treatment efficacy

## Abstract

**Background**: In violent communities, social rejection as a person with victim–offender attributes is associated with more intense symptoms of posttraumatic stress disorder (PTSD) and a higher propensity towards violence, i.e. appetitive aggression. Successful community reintegration encompassing adequate social acknowledgment of individuals with both a history of violence exposure and perpetration may be necessary to enhance the treatment effects of interventions addressing PTSD and aggression.

**Objective**: In this study, the effects of treatment and post-treatment traumatic events, violent offenses, and social acknowledgment (with sub-dimensions of general disapproval, family disapproval, and recognition as a person with both a history of violence exposure and commission) on changes in PTSD symptom severity and appetitive aggression from baseline to 8-month follow-up were investigated.

**Method**: Data were collected from 54 males recruited through a Cape Town offender reintegration programme for an intervention study targeting trauma and aggression (*n =* 28 treatment; *n =* 26 wait-list). Changes in PTSD symptom severity after treatment were assessed with the PTSD Symptom Scale-Interview, changes in appetitive aggression with the Appetitive Aggression Scale (AAS), post-treatment traumatic events with an adapted version of the Child’s Exposure to Violence Checklist, offenses with an adapted checklist from the AAS, and social acknowledgment with an adapted form of the Social Acknowledgment Questionnaire.

**Results**: Path analyses revealed negative relationships between ongoing societal disapproval and changes in PTSD symptom severity and appetitive aggression at 8-months, controlling for age. All other variables were non-significant, except for treatment, which was associated with PTSD symptom reduction.

**Conclusions**: As a complementary strategy to effective psychotherapeutic treatment, increased social acknowledgment may contribute significantly to the alleviation of PTSD symptoms and appetitive aggression. Psychological interventions should, therefore, not neglect the impact of societal factors on treatment effects.

## Abstract (es)

Planteamiento: En comunidades violentas, el rechazo social como persona con atributos de víctima-agresor se asocia con síntomas más intensos del trastorno por estrés postraumático (TEPT) y una mayor propensión a la violencia, es decir, a la apetencia por la agresión. Puede que sea necesaria una reintegración con éxito en la comunidad –que incluya un adecuado reconocimiento social de aquellos individuos con una historia tanto de exposición a la violencia como de agresión– para mejorar los efectos del tratamiento de las intervenciones que abordan el TEPT y la agresión.

Objetivo: En este estudio, se investigaron los efectos del tratamiento y los eventos traumáticos post-tratamiento, los delitos violentos y el reconocimiento social (con subdimensiones de desaprobación general, desaprobación familiar y reconocimiento como persona con antecedentes tanto de exposición a la violencia como de perpetración) sobre los cambios en la gravedad de los síntomas del TEPT y la apetencia por la agresión desde el inicio hasta el seguimiento a los 8 meses.

Método: Se recogieron datos de 54 varones reclutados a través de un programa de reintegración de delincuentes de Ciudad del Cabo para un estudio de intervención dirigido a trauma y agresión (*n =* 28 tratamiento, *n =* 26 lista de espera). Se evaluaron los cambios en la gravedad de los síntomas del TEPT después del tratamiento con la Escala-Entrevista de Síntomas de TEPT (*PTSD Symptom Scale-Interview*), los cambios en la apetencia por la agresión con la Escala de Apetencia por la Agresión (AAS, siglas en ingles de *Appetitive Aggression Scale*), los eventos traumáticos posteriores al tratamiento con una versión adaptada de la Lista de verificación de exposición a la violencia para niños (*Child’s Exposure to Violence Checklist)*, las agresiones con una lista de verificación adaptada del AAS, y el reconocimiento social con una forma adaptada del Cuestionario de Reconocimiento Social (*Social Acknowledgment Questionnaire*).

Resultados: Los análisis de ruta revelaron relaciones negativas entre la desaprobación social continuada y los cambios en la gravedad de los síntomas del TEPT y la apetencia por la agresión a los 8 meses, controlando la edad. Todas las demás variables no fueron significativas, excepto por el tratamiento, que se asoció con la reducción de los síntomas de TEPT.

Conclusiones: Como estrategia complementaria a un tratamiento psicoterapéutico efectivo, el aumento del reconocimiento social puede contribuir significativamente al alivio de los síntomas del TEPT y la apetencia por la agresión. Por lo tanto, las intervenciones psicológicas no deben descuidar el impacto de los factores sociales sobre los efectos del tratamiento.

## Abstract (zh)

**背景：**在有暴力风险的社区里，对受害者-施害者的社会拒绝与更强烈的创伤后应激障碍（PTSD）症状和更明显的暴力倾向（例如欲求性攻击）相关联。成功的社区再融合包括对遭受暴力者和施暴者给予足够的社会承认，这对提高干预PTSD 和攻击的治疗效果是必要的。

**目标：**本研究中，考察治疗效果和治疗后的创伤事件、暴力侵犯和社会承认（分维度为：总体否认、家庭否认、个人遭受和施加暴力的历史），对从基线期到8个月的追踪过程中PTSD症状严重程度和欲求性攻击的影响。

**方法：**数据采集于开普敦侵犯者再融合项目中一个关于创伤和攻击的干预研究中的54名男性（治疗组n=28，等候名单组n=26）。治疗后的PTSD症状改变用PTSD症状量表-访谈（PTSD Symptom Scale-Interview）测查，欲求性攻击的改变使用欲求性量表（AAS）测量，使用修订版儿童暴力暴露清单来测查治疗后的创伤事件，使用修订版AAS测量侵犯（offenses），使用修订版社会承认问卷（Social Acknowledgment Questionnaire）测量社会承认。

**结果：**路径分析揭示控制了年龄之后，进行中的社会否认和8个月时PTSD症状严重程度改变与欲求性攻击呈现负相关。所有其它的变量都不显著，除了治疗和PTSD症状减轻相关。

**结论：**作为有效的心理治疗的补充，增加社会承认度可能可以显著促进PTSD 症状和欲求性攻击的减轻。因此，心理干预不应该忽视社会因素在治疗效果上的影响。

## 1. Background

Short-term interventions such as the *Narrative Exposure Therapy for Forensic Offender Rehabilitation* (FORNET) have shown promising results in reducing both posttraumatic stress disorder (PTSD; Hermenau, Hecker, Schaal, Maedl, & Elbert, 2013; Köbach, Schaal, Hecker, & Elbert, 2015) and aggressive behaviour in victim–offender populations living in violent environments (Crombach & Elbert, 2015). Effective psychological treatment may be the core facilitator of such reductions in PTSD and the propensity toward violence – also known as *appetitive aggression* (Elbert, Weierstall, & Schauer, 2010) – in these populations. However, other non-psychological factors may mediate or hinder treatment effects and thus need to be considered. Recent research has demonstrated that the *social acknowledgment* of a person’s past violent experiences is significantly associated with PTSD and appetitive aggression in victim–offender populations and may represent a key environmental factor at a societal level (Author et al., 2017b). This study examined the association between such acknowledgment and treatment outcomes following a therapeutic psychological intervention.

In the literature, social acknowledgment is defined as a subtype of social support, referring not only to the emotional and instrumental support provided to an affected person by the immediate environment, but also to potential feelings of rejection and exclusion from the broader society because of one’s violent past (Maercker & Müller, 2004). The term covers the three constructs of *general disapproval* and *family disapproval*, i.e. the invalidation of one’s violent experiences or rejection by the social environment or family, and *recognition*, i.e. the perception that one’s suffering is acknowledged and understood. Research has shown that general disapproval is associated with more severe PTSD symptoms (Sommer et al.,, 2017a, 2017b; Jones, Mueller & Maercker, 2006; Mueller, Orth, Wang, & Maercker, 2009). In relation to family disapproval and recognition, the findings are more diverse, with results indicating that family disapproval is either positively associated with (Maercker & Müller, 2004) or unrelated to PTSD (Sommer et al., 2017b; Jones et al., 2006). Recognition has been revealed to be either negatively related to (Maercker, Povilonyte, Lianova, & Pöhlmann, 2009) or unrelated to PTSD (Mueller, Moergeli, & Maercker, 2008); however, in a South African victim–offender population, a positive relationship between recognition and PTSD was found, as discussed in Sommer et al. (2017b).

Recently – and in line with research demonstrating that social exclusion and rejection are associated with aggressive behaviour (DeWall, Twenge, Gitter, & Baumeister, 2009; Twenge, Baumeister, Tice, & Stucke, 2001) – Sommer et al. (2017b) found a positive association between general disapproval and appetitive aggression, a type of aggression which is perceived as self-rewarding rather than reactive (Elbert, Moran, & Schauer, 2017) and thus potentially fuels the cycle of violence in already violent environments such as low-income communities in South Africa. In contrast, recognition and family disapproval were found to be unrelated to appetitive aggression. Thus, general disapproval of violent experiences may influence not only the course of PTSD (Maercker & Horn, 2013) but also the trajectory of appetitive aggression, thereby impacting treatment outcomes.

This evidence notwithstanding, only a few studies have thus far investigated the effects of social factors during or after therapy on changes in PTSD. Tarrier and colleagues have demonstrated that negative social relationships and a lack of social support are related to poorer treatment outcomes in PTSD patients (Tarrier, Sommerfield, & Pilgrim, 1999; Tarrier, Sommerfield, Pilgrim, & Faragher, 2000). Furthermore, improvements in social acknowledgment through an intervention incorporating techniques from Cognitive Behavioural Therapy (CBT) have been shown to mediate the reduction of PTSD in trauma victims after treatment (Xu et al., 2015). With regard to changes in appetitive aggression, scores decreased over time in a treatment trial in Congolese soldiers who received social support via a reintegration programme (Hermenau et al., 2013). However, the associations between social acknowledgment and changes in appetitive aggression have yet to be studied.

In conflict areas such as low-income communities in South Africa, where residents are constantly exposed to severe forms of community violence (Eagle & Kaminer, 2013), factors beyond social acknowledgment that can potentially influence changes in PTSD and appetitive aggression must be acknowledged, as we address in this study. For example, post-treatment traumatic events that occur in between two measuring points in longitudinal assessments may be associated with changes in PTSD symptoms, potentially reinforcing the trauma memory and decreasing the likelihood of overall improvement. In fact, in a psychological treatment trial (Steketee, 1993), the stress response associated with *intermediate traumatic events* after treatment was found to be significantly related to relapse.

Regarding appetitive aggression, Elbert et al. (2010) suggest that violent environments (i.e. surroundings with many potential traumatic events) could be a breeding ground for appetitive aggression, as confirmed by a positive association between traumatic events and appetitive aggression (Sommer et al., 2017b). Furthermore, as violence perpetration increases, aggression may be perceived more positively (Sommer et al., 2017b; Köbach et al., 2015; Weierstall et al., 2013). As a result, newly experienced traumatic events and newly committed offenses may rekindle an individual’s ‘addiction to violence’ (Hecker, Hermenau, Crombach, & Elbert, 2015) following the completion of a psychological treatment programme and thereby decrease the likelihood of a reduction in appetitive aggression. This is especially relevant in South African contexts, where gang membership increases the probability of further violence perpetration (Jewkes et al., 2006) and victimisation.

Finally, changes in PTSD may be related to changes in appetitive aggression, due to the interrelatedness of these two states. The perpetration of violence often co-presents with victimisation (Malik, Sorensen, & Aneshensel, 1997), and PTSD has been shown to be related to aggression (Dyer et al., 2009; Jakupcak et al., 2007). This association may result from an underlying dysregulation of emotion (Weiss, Tull, Viana, Anestis, & Gratz, 2012) and impulse which may cause aggression, or individuals who suffer from numbing symptoms related to PTSD may utilise aggression in order to up-regulate themselves. Aggressive behaviour may thus serve as a coping mechanism when individuals lack other skills to handle traumatic situations (Spaccarelli, Coatsworth, & Bowden, 1995), resulting in a ‘victim-to-victimiser cycle’ (Glasser et al., 2001). This ‘fight’ reaction, which is often seen in PTSD patients (*DSM-5*; American Psychiatric Association, 2013), may be reflected in appetitive aggression to some extent, and reductions in PTSD may lead to reductions in appetitive aggression.

## 2. Objective

The hypotheses for this study are based on recent findings on associations between social acknowledgment, PTSD, and aggressive behaviour in a victim–offender sample of 290 South African males, published in Sommer et al. (2017b). A sample of juvenile ex-offenders was assessed prior to and eight months after treatment as part of an intervention trial comparing FORNET, CBT, and a wait-list control group. The hypotheses are as follows: There are (1) negative relationships between both intermediate traumatic events and general disapproval and changes in PTSD and, in turn, positive associations between both treatment and recognition and changes in PTSD; (2) negative relationships between intermediate traumatic events, offenses, and general disapproval and changes in appetitive aggression and, in turn, a positive association between treatment and changes in appetitive aggression; and (3) a positive relationship between changes in PTSD and changes in appetitive aggression. Due to a lack of available literature, no specific hypotheses were formulated for potential interaction effects with treatment.

## 3. Method

### 3.1. Participants and design

Between 2013 and 2014, 405 South African males were recruited through a reintegration centre for offenders and youth deemed to be at risk of experiencing and perpetrating violence due to high levels of gang violence and substance abuse present in the low-income communities of Cape Town in which the participants lived. After screening, 89 participants met the inclusion criteria (≥ 8 points on the PTSD Symptom Scale-Interview and ≥ 9 points on the Appetitive Aggression Scale) for the intervention study, which was conducted in several three-week-long camps. The 35 participants who were unable to attend the camp were assigned to the control condition ‘wait-list no camp’. All other participants were randomly assigned to either a treatment condition targeting trauma and aggression (eight FORNET sessions or seven CBT ‘Thinking for a Change’ sessions; about two hours/session) or a control condition (‘wait-list camp’). In the post-treatment assessment, PTSD symptom severity was significantly reduced in the FORNET treatment condition compared to the wait-list control condition. Scores for appetitive aggression and committed offense types did not significantly change in any of the treatment conditions. Further information on the treatments, therapists, and outcomes of the intervention study is detailed elsewhere (see Hinsberger et al., 2016).

The present study included 54 participants who were assigned to the treatment trial and who participated in at least one of two post-treatment assessments (8.1 and 17.7 months post-treatment). Participants were Black Xhosa-speaking males from low-income areas in Cape Town, South Africa, aged 14–40 years (*M* = 22.3, *SD *= 4.8). At baseline, 59% of participants were currently attending or had previously attended a reintegration programme, whereas 41% had not. Participants had attended school for 1–16 years (*M* = 10.26, *SD *= 2.06). To control for the impact of the treatment condition, a dummy variable was included in the path analyses, with those receiving treatment (FORNET/CBT) coded as ‘1’ (*n =* 28) and those in the control condition as ‘0’ (*n =* 26).

### 3.2. Materials

After the interviewers were trained in the concepts of trauma and aggression, individual structured interviews (translated from English to isiXhosa and back) were conducted by German and South African mental health experts supported by local interpreters. Participants were followed-up with the same interview schedule being used pre- and post-treatment. Interviewers were blind to experimental conditions.

#### 3.2.1. Intermediate traumatic event types

Traumatic event types occurring between pre- and post-assessment were explored using an adapted version of the Child’s Exposure to Violence Checklist (CEVC; Amaya-Jackson, 1998), comprising 36 items on the direct experiencing (e.g. ‘Have you been attacked with a weapon by a family member?’) and witnessing (e.g. ‘Have you seen someone being killed?’) of potentially traumatic events. Items were rated dichotomously: 1 if the event had been experienced, 0 if not. This measure has excellent psychometric properties (Fincham et al., 2009) and has previously been administered in South African high-risk males (Hinsberger et al., 2016). Kuder-Richardson’s alpha in this sample was .86. Traumatic event types were summed with a possible range from 0 to 36 points.

#### 3.2.2. PTSD symptom severity

PTSD symptom severity during the past two weeks was assessed with the PTSD Symptom Scale-Interview (Foa & Tolin, 2000), with each of the 17 items rated from 0 (*not at all*) to 3 (*very much*). The scale indicates the extent to which the index trauma evoked B (re-experiencing), C (numbing/avoidance), and/or D (hyper-arousal) PTSD symptoms from the *Diagnostic and Statistical Manual of Mental Disorders* (4th ed., text rev.; *DSM-IV-TR;* American Psychiatric Association, 2000). Studies have confirmed the measure’s usefulness in terms of its psychometric properties in South African at-risk youth (Sommer et al., 2017a). Items were summed with a possible range from 0 to 51 points.

#### 3.2.3. Appetitive aggression

Attraction to violence was evaluated using the Appetitive Aggression Scale (AAS; Weierstall & Elbert, 2011), which has previously been administered in South African high-risk males (Hinsberger et al., 2016). This questionnaire contains 15 statements (e.g. ‘Is it fun to prepare yourself for fighting?’) to be rated from 0 (*I totally disagree*) to 4 (*I totally agree*) according to the respondent’s current point of view. Items were summed with a possible range from 0 to 60 points.

#### 3.2.4. Intermediate offense types

Participants were asked to indicate whether they had committed any of 21 offense types from the AAS in the past six months (e.g. Have you injured another person with a weapon [e.g. a knife] in the past six months?”). This measure’s validity has been confirmed in at-risk South African youth (Sommer et al., 2017b). Kuder-Richardson’s alpha in this sample was .91. Offense types were summed with a possible range from 0 to 21 points.

#### 3.2.5. Social acknowledgement

The 16 items on the Social Acknowledgement Questionnaire (SAQ; Maercker & Müller, 2004), rated from 0 (*not at all*) to 3 (*completely*), examine the perception of recognition or disapproval from one’s family and society after traumatic events. A version adapted to participants in a victim–offender population has been successfully administered in a South African sample (Sommer et al., 2017b). Of the three subscales, *recognition* captures positive aspects (e.g. ‘The reactions of my acquaintances were helpful’), whereas *general disapproval* (e.g. ‘Most people cannot understand what I went through’) and *family disapproval* (e.g. ‘My experiences are underestimated by my family’) reflect negative aspects of social acknowledgment. The item ‘My boss showed full understanding for any absence from work’ was adapted (possible answer: *not applicable*) to account for the extremely high unemployment rate of Black Africans in South Africa (Statistics South Africa, 2016). The recognition score, which included the critical item, was thus divided either by 5 or 6, based on whether the item was rated or not. Cronbach’s alpha in this sample was: recognition, α = .59; general disapproval, α = .73; family disapproval, α = .57. Items were summed for each subscale with a possible range from 0 to 3 points for recognition and 0 to 15 points for general disapproval and family disapproval.

### 3.3. Procedure

Participants were invited to take part in the intervention trial and the follow-up visits with support from the above-mentioned reintegration centre. Trained German and South African therapists offered individual treatment within the frame of the intervention study. Ethical approval was obtained from the Ethical Review Boards of the University of Konstanz and the University of Cape Town and from the Health Research Ethics Committee of Stellenbosch University; clinical trials registration ID: NCT02012738. Participants gave informed consent before the initial assessment (for minors, this was provided by parents/caretakers) and received financial compensation of 100 South African Rand (ZAR) per (follow-up) Interview (minimum hourly wage for full-time domestic workers: ZAR 10.59; South African Department of Labour, 2014).

### 3.4. Data analyses

The data analyses focused on the first post-treatment assessment, in which 39 participants took part; in order to include all 54 participants, missing values were estimated using maximum likelihood estimation on the basis of all the available data on the participants. Differences between treatment conditions with regard to all pre- and post-treatment variables relevant to the path analyses were tested with Mann-Whitney U-tests. Correlations between all variables included in the hypothesised path model were calculated using SPSS 21. Path analyses were conducted using AMOS 23. Intermediate traumatic events, offenses, general disapproval, recognition, and treatment represented predictors; changes in PTSD and appetitive aggression were considered outcomes. Model fit indices were the chi-square statistic (χ^2^), which should be non-significant, the comparative fit index (CFI), and root mean square error of approximation (RMSEA).

## 4. Results

### 4.1. Group comparisons and correlations

Based on complete data for 39 participants, there were no significant differences between conditions with regard to any of the pre-treatment and post-treatment variables relevant to the present model (*p *> .05, Mann-Whitney U-test), except for changes in PTSD symptoms (*p* = .010), for which significantly greater improvement was found in the treatment condition (*M* = 8.23, *SD *= 13.41) than in the control condition (*M* = −2.84, *SD *= 12.12). There was a significant variance in age (*p* = .010) between the treatment (*M* = 23.64, *SD *= 4.68) and control conditions (*M* = 20.89, *SD *= 4.53), and age was consequently included in the model.

Correlations between predictors and outcomes are shown in Table 1. Because family disapproval exhibited a significant correlation with changes in appetitive aggression, it was added as a control variable in the path analyses.

**Table 1.** Means, standard deviations (*SD*), and correlations (Spearman’s rho) between predictors and changes in PTSD symptom severity and appetitive aggression.

Variable	Mean (*SD*) [range]	PSS-I change score	AAS change score
Intermediate			
traumatic event types	11.71 (6.09) [0–27]	−.41*	−.13
offense types	8.08 (4.90) [1–16]	−.23	−.35*
SAQ recognition	1.69 (.69) [0–3]	−.31	−.29
SAQ general disapproval	8.62 (3.69) [0–15]	−.48**	−.47**
SAQ family disapproval	6.85 (3.91) [0–14]	−.24	−.38*
Treatment/Control1	–	.38*	−.09
PSS-I change score	5.54 (13.88) [21–29]	1	.33*
AAS change score	3.72 (13.88) [33–39]	.33*	1

PSS-I = PTSD Symptom Scale-Interview, AAS = Appetitive Aggression Scale, SAQ = Social Acknowledgment Questionnaire. Change scores resulted from subtracting the post- from the pre-treatment score, such that a positive score represents symptom reduction. *p < .05, **p < .01 (two-tailed), 1Point-Biserial Correlation Coefficient

### 4.2. Path analyses

**Figure 1.**
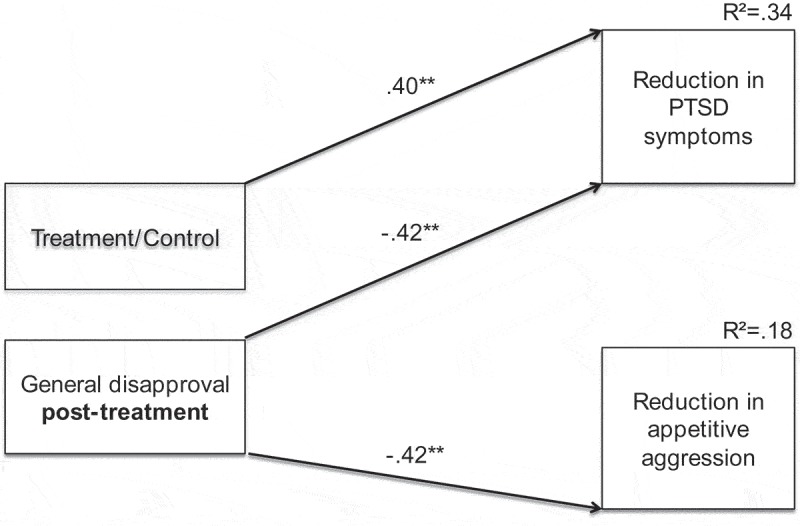
Path model of relationships between general disapproval post-treatment, treatment (1) vs. control (0), and changes in PTSD symptom severity and appetitive aggression. Paths with arrowheads indicate directed associations. Standardised regression weights are shown. **p < .01.

Fit statistics for our hypothesised model were adequate: χ^2^ (2, *N *= 54) = 2.67, *p *= .263, CFI = .978, RMSEA = .080 (90% confidence interval [CI] = .000–.296). Paths from traumatic events to changes in PTSD and appetitive aggression, from offenses to changes in appetitive aggression, and from recognition to changes in PTSD were non-significant. The path from age to changes in appetitive aggression showed a negative trend (*p *= .054). Non-significant paths and the remaining non-significant inter-correlation between treatment and general disapproval were excluded. The revised model is shown in Figure 1.

Adding family disapproval as another control variable did not significantly increase the explained variance, neither in the full model nor in the final model. For validation purposes, the hypothesised model was also calculated for the 39 participants with complete data, resulting in the same paths as described in the final model, with nearly identical fit values: χ^2^ (3, *N *= 39) = 2.78, *p *= .426, CFI = 1.000, RMSEA = .000 (90% CI = .000–.266).

The final model accounted for 34% of the variance in reduction in PTSD and 18% for appetitive aggression. Considering the norms of a good-fitting model (Hu & Bentler, 1999), fit statistics were excellent: χ^2^ (3, *N *= 54) = 2.80, *p *= .423, CFI = 1.000, RMSEA = .000 (90% CI = .000–.226). The treatment condition exhibited greater changes in PTSD than the control condition (b = 11.08). General disapproval was negatively related to changes in both PTSD (b = −1.61) and appetitive aggression (b = −1.59).

Raw scores for PTSD severity ranged from 8 to 37 pre-treatment (*M* = 18.96, *SD* = 7.83) and from 0 to 39 post-treatment (*M* = 14.62, *SD* = 12.46), and those of appetitive aggression from 9 to 52 pre-treatment (*M* = 25.78, *SD* = 11.83) and from 0 to 47 post-treatment (*M* = 24.00, *SD* = 15.17). Consequently, the final model’s results indicate that although the means of both outcomes decreased after treatment, general disapproval impeded further changes in PTSD and appetitive aggression.

## 5. Discussion

This study investigated potential associations between changes in PTSD and appetitive aggression and intermediate traumatic events, offenses, general disapproval, recognition, and treatment condition from pre-treatment to post-treatment in high-risk individuals from poor communities in Cape Town. Consistent with results from Sommer et al. (2017b), general disapproval was not only related to more severe PTSD symptoms (Forstmeier, Kuwert, Spitzer, Freyberger, & Maercker, 2009) but also negatively related to changes in PTSD after treatment and over time. This is in line with findings by Xu et al. (2015) indicating that a positive change in social acknowledgment is associated with reduced PTSD after treatment. Similarly, Nietlisbach and Maercker (2009) conclude that PTSD patients exhibit stronger negative responses to social exclusion than healthy individuals, which is linked to the exacerbation of mental impairments.

General disapproval was also negatively associated with changes in appetitive aggression, which may indicate that feeling excluded from society could impede a possible reduction in appetitive aggression. Ex-gang members may experience rejection for their previous offenses; because such social exclusion could discourage them about the chances of making positive changes in their lives (Dorkins & Adshead, 2011), the environment of ex-offenders may influence their ability to abstain from violence (Serin & Lloyd, 2009).

The treatment condition exhibited greater changes in PTSD than the control condition (b = 11.08), indicating that trauma- and aggression-focused treatment can significantly reduce PTSD in South African victim–offender populations. With regard to changes in appetitive aggression, no significant relationship with treatment was found. This may be an age effect (participants in the treatment condition were older than those in the control condition), as changes in appetitive aggression were negatively correlated with age. However, including the interaction between treatment and age as a predictor of change in appetitive aggression did not significantly increase the explained variance in the final model.

Recognition from one’s environment was not significantly related to changes in PTSD. This is in line with research indicating that positive social reactions are not as influential as negative ones (Jones et al., 2006). Although intermediate traumatic events were negatively related to changes in PTSD in the correlation analysis, this relationship was non-significant in the overall model. The same holds true for the path from intermediate offenses to changes in appetitive aggression. One explanation for these findings could be that our data provided too little power; it is also possible that general disapproval ruled out other intervening variables.

The path from changes in PTSD to changes in appetitive aggression indicated a positive trend (*p *= .091), but a non-significant one. This result suggests that the alleviation of PTSD symptoms may not necessarily reduce the drive for appetitive aggression, which is promoted when moral inhibitions collapse, as may occur under extremely violent conditions (Elbert et al., 2010). If these moral inhibitions cannot be resurrected due to factors such as general disapproval – implying exclusion from society and thus an obstacle to reconnecting with its values (Dorkins & Adshead, 2011) – the restored psychosocial functioning may even lead to more violence perpetration.

A larger sample size might have allowed more complex mapping in the proposed statistical models and thus greater statistical power. Tracking participants within South African victim–offender populations is challenging, as participants may be homeless, frequently intoxicated, or in hiding due to gang-fights and thus inaccessible for follow-ups. When asked about perpetrated events, participants may have provided socially desirable answers, potentially increasing the error variance of the data. Furthermore, the Social Acknowledgment Questionnaire measures subjective perceptions, which may differ to some extent from objective social interactions (Maercker & Müller, 2004) as PTSD symptoms may potentially bias a person’s perception of social support (Charuvastra & Cloitre, 2008). Because this measure only assesses three domains of social acknowledgment, it may not capture all the cultural nuances of this construct. Finally, as correlation does not imply causation, causal inferences should be drawn with caution.

## 6. Conclusion

With respect to intervention approaches, a focus only on the individual and only on the trauma can neglect external factors that may influence treatment outcomes, contributing to the limited effectiveness of individual psychotherapeutic approaches for PTSD (Maercker & Hecker, 2016). Social influences should thus be considered when treating PTSD (Maercker & Horn, 2013), and effective community-wide interventions are needed to improve social support (Kaniasty & Norris, 2008), which has been shown to be essential in the successful reduction of appetitive aggression in combination with interventions like FORNET (Hermenau et al., 2013). Conversely, the lack of social support may contribute to environments that promote reoffending (Payne, Tewksbury, & Mustaine, 2010). Acknowledging ex-offenders instead of excluding them from society may lower appetitive aggression. Narratives in which offenders try to integrate their victimisation and perpetration into a complete picture (Dorkins & Adshead, 2011) may represent one approach intended to promote understanding when shared with the community.

In the attempt to ensure sustainable psychological intervention effects in offender populations, it should be stressed that social acknowledgment – in particular, societal disapproval related to past violent experiences as both perpetrator and victim – may represent an important social factor that must be addressed in the treatment of PTSD and appetitive aggression. In addition to individual therapeutic interventions, the affected community should be targeted in the holistic management of offenders.
